# The challenges of dynamic vulnerability and how to assess it

**DOI:** 10.1016/j.isci.2022.104720

**Published:** 2022-07-04

**Authors:** Marleen C. de Ruiter, Anne F. van Loon

**Affiliations:** 1 Institute for Environmental Studies (IVM), Vrije Universiteit Amsterdam, Amsterdam, the Netherlands

**Keywords:** Earth sciences, Environmental event, Social sciences

## Abstract

Recent disasters have demonstrated the challenges faced by society as a result of the increasing complexity of disaster risk. In this perspective article, we discuss the complex interactions between hazards and vulnerability and suggest methodological approaches to assess and include dynamics of vulnerability in our risk assessments, learning from the compound and multi-hazard, socio-hydrology, and socio-ecological research communities. We argue for a changed perspective, starting with the circumstances that determine dynamic vulnerability. We identify three types of dynamics of vulnerability: (1) the underlying dynamics of vulnerability, (2) changes in vulnerability during long-lasting disasters, and (3) changes in vulnerability during compounding disasters and societal shocks. We conclude that there is great potential to capture the dynamics of vulnerability using qualitative and model-based methods, both for reproducing historic and projecting future dynamics of vulnerability. We provide examples using narratives, agent-based models, and system dynamics.

## Graphical abstract

**Figure:**
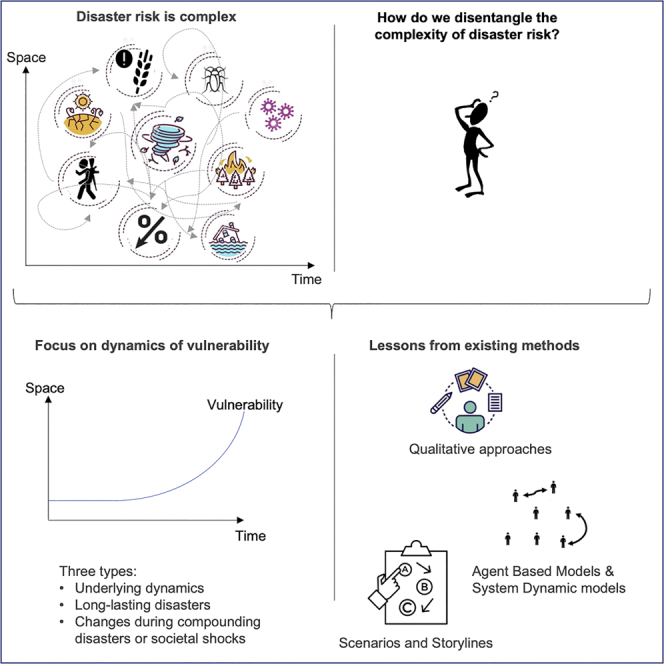

## Introduction

Recent disasters have demonstrated the challenges faced by society as a result of the increasing complexity of disaster risk, some examples of which are discussed in Box 1. A country or region hit by a natural hazard while still recovering from the impacts of an earlier hazard faces many different challenges than when it is hit by a single hazard that occurs in isolation. Alternatively, the co-occurrence of hazards or hazard drivers, also known as compound hazards (Leonard et al., 2014; Zscheischler et al., 2018), can also aggravate impacts. In this perspective article, we define risk as the product of hazard, exposure, and vulnerability where a hazard is a phenomenon that causes impact to exposure, assets, and people in harm’s way, depending on their vulnerability. Vulnerability is defined as people, assets, or a system’s susceptibility to the impacts of hazards and is determined by physical, social, economic, and environmental vulnerability factors or processes, such as available resources and assets, food security, and coping capacity (UNDRR, 2020). In this article, we focus on vulnerability from a disaster risk perspective.

Box 1 **Examples of past disasters, demonstrating the complexities of disaster risk**

**Figure 1:**
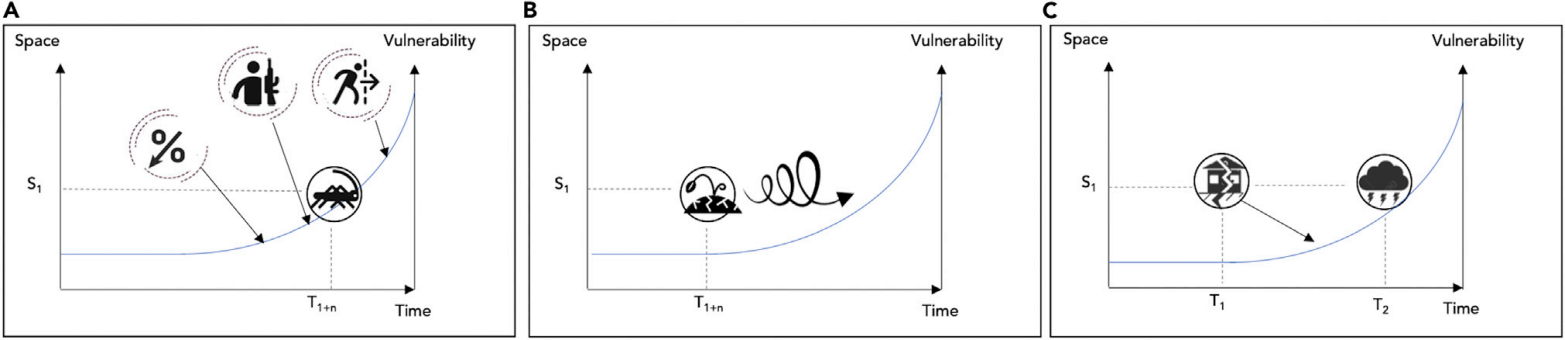
Three key types of dynamics of vulnerability The panels show from left to right: underlying dynamics of vulnerability, such as (internal) migration, conflicts, or economic recession (A); dynamics during long-lasting vulnerability, such as effects of (mal)adaptation, eroding financial resources, or mental well-being (B); and dynamics of vulnerability owing to consecutive or compound disasters, such as the effects of an earlier hazard on the vulnerability at the time of a second hazard (C).

Super typhoon Goni (locally known as super typhoon Rolly) is considered the strongest recorded tropical cyclone to ever make landfall and it hit the Philippines in November 2020, in the midst of the COVID-19 pandemic (Rocha et al., 2021). An estimated 68.8 million people were affected by typhoon Goni. The ongoing pandemic impacted people’s ability to coop with the impacts of the typhoon, which in turn triggered floods and landslides. COVID numbers surged owing to overcrowding in evacuation centers and the limited ability to observe social distancing regulations. Aid and recovery efforts were significantly challenged by the need to observe COVID-19 health precautions (Gonzalo Ladera and Tiemroth, 2021; IFRC-DREF, 2020). With many low-income Filipinos living in already more vulnerable low-lying coastal areas (Morin et al., 2016) and the ongoing pandemic increasing people’s vulnerability (Zuñiga and Villoria, 2021), Roche et al. (2021) demonstrate that the impacts of the typhoon, which caused homelessness and loss of access to basic amenities, further exacerbated vulnerability. This dynamic of vulnerability is also demonstrated in Figure 1A.

In spring 2019 cyclones Idai and Kenneth hit the coast of Mozambique only six weeks apart, causing hundreds of fatalities and displacing 200,000 people. After cyclone Idai, local financial resources were strained, impairing the response to the impacts of subsequent cyclone Kenneth (Emerton et al., 2020). This in turn forced people to remain in the affected area. The prolonged exposure to standing water contributed to a subsequent cholera outbreak (Cambaza et al., 2019). In January 2021, Mozambique was once again hit by a cyclone (cyclone Eloise), while recovery from the 2019 events was still underway: 45% of the families who were living in resettlement sites owing to cyclone Idai were affected by cyclone Eloise (International Organization for Migration (UN-IOM, 2020)). This is one of the many examples showing that the occurrence of consecutive disasters can severely aggravate the impacts of disasters, both by the consecutive nature of the hazards themselves, as well as spatiotemporal dynamics in exposure and vulnerability caused by the earlier events. This dynamic of vulnerability is also demonstrated in Figure 1C.

East Africa experienced a series of drought and flood events between 2016 and 2018 that coincided with a number of anthropogenic and biological hazards. The combination of drought and crop pests led to displacement, which together with political instability exacerbated ethnic conflicts, food insecurity, and health issues, which in turn increased vulnerability to subsequent dry seasons and flooding (Matano et al., in review). This led to a large humanitarian disaster in the region, with 4 million people under food insecurity in Kenya (FEWS NET, 2018) and 8 million in Ethiopia (FEWS NET, 2019). More recently, in 2020-22, floods hit a region already vulnerable owing to a desert locust invasion, the COVID-19 pandemic, and conflicts, destabilizing the economy. The review by Kassegn and Endris (2021) points out long-term changes in vulnerability factors such as GDP (3% decrease) and states that “the threats do not just have short-term impacts on socio-economic conditions of the people in the region in general and in Ethiopia in particular, they exacerbate prevailing food insecurity and undermine livelihoods and development gains that took years to build.” (Kassegn and Endris, 2021, p.1). This dynamic of vulnerability is also demonstrated in Figure 1C.

In 2018 Cape Town was very close to Day Zero, when there would no longer be water from the tap for its four million inhabitants (Ziervogel, 2019; Savelli et al., 2021). The water shortage was owing to a combination of several years of below-normal rainfall, but impacts were felt unequally throughout the city. Unsustainable use of water by the upper and middle class had exacerbated the water shortage (Enqvist and Ziervogel, 2019). During the water crisis, these high-water users had to cut back their water use by a lot, but also had most coping strategies (e.g., drilling private groundwater wells; e.g. Simpson et al., 2020). People living in townships, already vulnerable, saw an aggressive metering campaign, restricting water access even for basic sanitation and growing family food, and a massive increase in the price of water, eating into financial resources (Enqvist et al., 2022). After the drought inequalities had increased, the upper and middle class had reduced vulnerability because of more access to water sources, and the lower class had increased vulnerability because of less access to water and lower financial resources (Savelli et al., 2021; Enqvist et al., 2022). This dynamic of vulnerability is also demonstrated in Figure 1B.

In recent years, international agreements such as the Paris Agreement and the UN’s Sendai Framework for Disaster Risk Reduction (SFDRR) have called upon the science community to move away from so-called hazard-silo thinking and improve our understanding of the complexities of disaster risk (UNDRR, 2015). In the hazard-silo thinking paradigm, the risk is typically represented as spatiotemporally static and traditionally focuses on the risk of one hazard (de Ruiter et al., 2021a; 2021b). The SFDRR (2015) gave a boost to research looking into the complexities of disaster risk, with a strong push to account for spatiotemporal dynamics of risk and risk components. Many recently published research and perspective papers, and policy reports have underscored the need for a more comprehensive understanding of multi-hazard risk (Global Risk Assessment Framework, 2019; Reichstein et al., 2021; UNDRR, 2019) and attempted to address this call. The attempt in recent years to increase our understanding of multi-hazard risk is demonstrated by the recent surge in compound-hazard-related publications: a google scholar search shows that since 2015 (the year of the implementation of the SFDRR), 236 papers have been published mentioning “compound hazard.” Several studies have focused on assessing the co-occurrence of climate drivers and hazards, commonly defined as compound events (Cutter, 2018; Leonard et al., 2014; Ridder et al., 2020; Zscheischler et al., 2018). These studies tend to focus on one hazard type and the co-occurrence of its different (climate) drivers, for example, floods caused by the co-occurrence of high discharge, storm surge, and/or precipitation (e.g., Couasnon et al., 2020; Mazdiyasni and AghaKouchak, 2015; Paprotny et al., 2020; Moftakhari et al., 2019; Wahl et al., 2015) or joint dry hazards such as droughts, heatwaves, and fires (Matthews and Marston, 2019; Raymond et al., 2020b; Sutanto et al., 2020; Zscheischler and Seneviratne, 2017). Recently, some studies assessed the compound occurrence of different types of climate drivers and hazards (Ridder et al., 2020).

However, in disaster risk assessments, an often-overlooked component continues to be that of underlying societal circumstances and their dynamics (e.g. Drakes and Tate, 2022; Hagenlocher et al., 2019; Simpson et al., 2021). As Raju et al. (2022) stated: “Disasters occur when hazards meet vulnerability.” This aspect of vulnerability encompasses a range of social, economic, and political processes. The examples of the Philippines and East Africa (Box 1) demonstrate how the COVID-19 pandemic coincided many times with other hazards in societies that already face many other social, political, and economic challenges, highlighting how social inequalities affect an ongoing disaster (Kelman, 2020). Recent studies have shown that the most vulnerable people are disproportionally more susceptible to the impacts of hazards compounding with the COVID-19 pandemic (Dargin et al., 2021; Kassegn and Endris, 2021; Kruczkiewicz et al., 2021; Lambert et al., 2020; Phillips et al., 2020). Furthermore, pre-existing issues and inequalities can be exacerbated by hazards, for example, a long-lasting drought accelerated the water crisis in Cape Town (Box 1; Enqvist and Ziervogel, 2019; Savelli et al., 2021). Some recent studies have come to recognize that in a coupled system it is difficult (if not impossible) to completely separate hazard and underlying processes (Hall and Leng, 2019), for example, in the case of an anthropogenic hazard, such as a conflict, and underlying fragility. This begs the question, of whether a conflict is an anthropogenic hazard or a social process that influences vulnerability to hazards or both? For droughts, this decoupling is particularly challenging, compared to other natural hazards, because droughts are complex long-duration hazards and the distinction between a drought and a non-drought event and the distinction between drought impacts and socio-economic changes with other drivers can be very unclear (AghaKouchak et al., 2021; Hall and Leng, 2019). In this article, we focus on long-term processes rather than sudden shocks such as conflict situations. We refer to the existing body of literature for more on anthropogenic hazards and their vulnerability (e.g., Ide et al., 2020; Peters, 2021; Xu et al., 2016).

Instead of taking a perspective where we start from the hazard, we argue for a changed perspective, starting with the circumstances that determine dynamic vulnerability. In this article, we refer to the dynamic aspects of vulnerability as “dynamics of vulnerability.” The dynamics of vulnerability, which are often excluded from dynamic risk assessments, are threefold: (1) the underlying dynamics of vulnerability, (2) changes in vulnerability during long-lasting disasters, and (3) changes in vulnerability during compounding disasters. The assessment and incorporation of these three dynamics of vulnerability into our risk assessments require different methods. We believe that, methodologically, lessons can be learned from methods developed in the fields of compound and multi-hazard risk, and socio-hydrology or socio-ecological systems.

In this perspective article, we aim to discuss the complex interactions between hazards and vulnerability and suggest methodological approaches to learning from the compound and multi-hazard, and socio-hydrology research communities. First, we provide a brief historic overview of major developments in the field of vulnerability research. Next, we discuss different dynamics of vulnerability and identify knowledge gaps. Finally, we propose possible methods from other sub-fields to improve our assessment of these dynamics and how these dynamics can be incorporated into multi-risk assessments.

## A brief history of vulnerability

We distinguish several key developments in the field of vulnerability research in recent decades. These include (1) a shift from a focus on physical vulnerability to including social vulnerability, (2) changes in common indicators used to measure vulnerability, and (3) most recently, a change from static toward dynamic vulnerability. Later in discussion, we provide a brief discussion of the history of vulnerability research to better understand where the field is at today and to identify knowledge gaps that continue to exist.

### Physical and social vulnerability

The vulnerability field has long faced the challenge of a lack of agreement on the meaning of vulnerability (Cutter and Finch, 2008). The physical component of vulnerability has traditionally been researched most (Notaro et al., 2014), where physical vulnerability is defined as the vulnerability of elements and assets such as buildings and infrastructure (Douglas, 2007). However, in 1983 already, Perrow (1983) pointed out that disasters happen in complex systems, and that interactions and feedback are an integral part of vulnerability. The author explained that vulnerability has a very strong influence on trickle-down effects, which can cause total cumulative impacts to be much larger. To understand loss potential (risk), it is crucial to understand the context in which a hazard takes place (Cutter et al., 2000). The concept of social vulnerability was introduced in the mid-nineties already and was defined as deriving from the circumstances of everyday life and their changes (Anderson 1995; Cutter et al., 1996; Hewitt 1997; Blaikie et al., 1994; Mileti 1999). Cutter (1996) pointed out the importance of accounting for the causes of changes in social vulnerability. Turner et al. (2003) discuss the limitations of two, at the time archetypal, models that informed vulnerability analysis: the Risk-Hazard (RH) model, which aims to understand the impacts of a hazard as a function of exposure and an implicit accounting for vulnerability, and the Pressure-and-Release (PAR) model, where there is an explicit emphasize on social conditions of exposure and vulnerability. The RH model does not account for the ways in which the system can alter the impacts of a hazard, nor does it account for the role of social structures and institutions in creating heterogeneity in exposure and circumstances. The PAR model does account for the differentiations of vulnerability using different indicators (e.g., ethnicity and gender) but does not account for the larger human-environment system (Turner et al., 2003).

### Vulnerability assessments

Modeling vulnerability has long been seen as the most challenging part of a risk assessment (Douglas, 2007). Turner et al. (2003) suggest a “reduced-form” vulnerability framework that aims to enable the assessment of vulnerability as a coupled human-environmental system (meaning the interaction between physical and social processes in relation to risk). The authors recognize that ideally, a comprehensive vulnerability analysis encompasses the complete system, but they deem this to be “unrealistic” owing to data challenges. Nonetheless, they underscore the need to perceive vulnerability as part of a larger system with linkages that act across different spatiotemporal scales. Adger (2006) observes several challenges in vulnerability research, namely that of finding a metric(s) that allows the quantification of vulnerability. This is mainly caused by the dynamic nature of vulnerability and challenges to understand and capture linkages and mechanism that influence vulnerability (Adger, 2006). Common vulnerability indicators included measurable characteristics of an entire country such as GDP (IPCC, 1995). However, owing to its complexity, it was argued that vulnerability cannot be measured by simply using one indicator (Adger, 2006; Cutter and Finch, 2008). Therefore, vulnerability was seen as a challenging component of risk to account for (how does one account for all probabilities and contingencies of a disaster and is this the same for different types of disasters) (Adger, 2006; Cutter, 2003). This led to the development of several vulnerability indices that allowed for the inclusion of multiple indicators such as the Social Vulnerability Index or SoVI (Cutter et al., 2003) and several others (we refer to De Ruiter et al., 2017 for an overview of common vulnerability indicators and assessment methods). The SoVI encompasses 85 different variables that characterize the different dimensions of social vulnerability (e.g., education, gender, renter versus homeowner, occupation, and so forth). The SoVI was applied first to the US where the SoVI indicators were brought back to 11 independent indicators (Cutter et al., 2003). The use of indices allowed for the assessment of vulnerability across space and time (Cutter and Finch, 2008). Cutter and Finch (2008) used their SoVI to assess spatial and temporal changes in vulnerability in the US, per country for four decades (1960–2000). Such assessments were complicated by a lack of data (both in terms of quality and accessibility) and conceptual shortcomings. Despite data challenges, in recent years, studies have provided global- or continental-scale vulnerability assessments for climate-related disasters (Formetta and Feyen, 2019), and for individual hazards such as droughts (e.g. Ahmadalipour and Moradkhani, 2018; Meza et al., 2020) and sea-level rise and coastal flooding (e.g. Kulp and Strauss, 2019), which are an important step forward. Another challenge raised is that of the discrepancy between objective vulnerability and how the vulnerable perceive their own vulnerability (Adger, 2006; Kasperson et al., 2005). Experienced vulnerability or (in)security is difficult to measure, highly culturally specific, and the impacts of a disaster on the perception of (in)security itself can be very difficult to assess (Adger, 2006).

### Increasing attention to the dynamics of vulnerability

As human-environment interactions are important in the development of disasters, vulnerability cannot be viewed as static and should be assessed in a dynamic way. The social sciences have long recognized this non-static nature of vulnerability (Collins, 2008; Cutter et al., 2000; Hewitt 1983; Oliver-Smith 2002; Savelli et al., 2022; Wisner and Luce 1993). Nonetheless, the dynamics of vulnerability have not been mainstreamed yet in disaster risk assessments. Hagenlocher et al. (2019) found that drought vulnerability and risk assessments rarely employ dynamic approaches. Also for other hazards such as floods, dynamic vulnerability approaches are rare (Moreira et al., 2021). Dynamic vulnerability is especially important in long-duration hazards, such as drought and pandemics, and in multi-hazard settings, in which one hazard can increase vulnerability to the next. Therefore, scientists have argued a need to understand risk dynamics across hazards (Gallina et al., 2016; Gill and Malamud, 2016). For example, measures implemented to address vulnerability to a particular hazard type can adversely influence vulnerability to another hazard (de Ruiter et al., 2021a, 2021b; Ward et al., 2020).

Next, several recent studies point out the need to quantify the dynamics between the three components of risk over time and space (Alves et al., 2020; Bevacqua et al., 2021; de Ruiter et al., 2020; Hagenlocher et al., 2019; Kuhla et al., 2021; Lin et al., 2020). A large part of recently published studies has focused on the dynamics of hazards and hazard drivers, both within science as well as in disaster risk management (Cutter, 2018; Raymond et al., 2020a; Scolobig et al., 2017). Simpson et al. (2021), for example, designed a framework to better capture the complexities of climate change risk and its drivers. Terti et al. (2015) developed a conceptual flash flood vulnerability model that aims to capture spatial and temporal dynamics of vulnerability such as the location of people during nighttime hours influencing their ability to respond proactively, but recognize existing challenges such as data availability and the limited scale at which an assessment reasonably can be conducted. In response to the traditional static approach to assessing social vulnerability, Fekete (2019) conducted an assessment of changing social vulnerability in Germany over a 10-year period using longitudinal demographic indicators of disaster risk, such as GDP, education, (un)employment, and migrants. For several indicators of social vulnerability such as intra-household differences in gender and age, Otto et al. (2017) assess thresholds of substantial change in human vulnerability caused by climate change. The authors acknowledge the need for more research and the development of new methodological approaches to assess interactions between different environmental and social drivers and their impacts on human well-being. Hagenlocher et al. (2019) also argue that a methodology for the assessment of dynamic vulnerability is urgently needed and that such an approach should account for non-linearities and feedback. However, a major challenge is posed by the data required to conduct such an analysis. Furthermore, the authors point out the existing lack of research that includes the validation of vulnerability assessments, which will be especially challenging for dynamic vulnerability assessments. Finally, within the field of emergency management and impact-based forecasting, the need for dynamic vulnerability data has been underscored (Harrison et al., 2022; Merz et al., 2020). Hence, there is a clear lack of and need for a comprehensive understanding of the dynamics of vulnerability and methodological approaches to assess these dynamics.

### Vulnerability as a dynamic, social process

In recent years, some disaster risk studies have called for a different paradigm in which disasters are perceived within the “bigger picture,” as part of broader, social processes such as dimensions of inequality, power relations, and the political economy of social resources (e.g., Balch et al., 2020; Moseley 2022; Raju et al., 2022; Rusca et al., 2021).

An important aspect of this changing perception, where we perceive disasters as complex systems, is the need to better understand human-environment interactions. For example, Simon (2014) describes vulnerability not only as continuously dynamic over time and space, but also as a process of interaction between landscapes and humans using and altering these landscapes. Within the field of socio-hydrology, attempts have been made to better understand the coupled human-water system (e.g., Di Baldassarre et al., 2013; Pande and Sivapalan, 2017). Rusca et al. (2021) developed an analytical approach to analyze the complexity of future extreme flood events and multi-scalar societal responses. They show that it is crucial to account for the heterogeneity in society’s vulnerability. For example, while Chile is on average not a low-/middle-income country it does have a large welfare gap so a large-scale indicator such as GDP does not explain impacts in lower income areas. The authors describe vulnerability as an integral part of disasters and what makes disasters human constructs (Rusca et al., 2021).

Chmutina et al. (2021) argue that the way in which the UN’s Sendai Framework for Disaster Risk Reduction (2015) assesses progress in terms of disaster risk reduction (DRR) remains hazard or disaster-focused instead of being rooted in vulnerability and development as root causes of risk, calling disasters “time-delayed manifestations of structural violence and maldevelopment.” We hypothesize that this is especially the case for hazards where more structural DRR measures can be taken, such as floods. For creeping hazards, such as droughts, there is a growing recognition that the assessment of vulnerability is crucial. However, Hagenlocher et al. (2019) showed with a systematic review of 105 papers that many conceptual and methodological gaps persist. Also, most papers in their selection focused only on the social dimension of vulnerability without taking into account system interactions (Hagenlocher et al., 2019).

## Key types of dynamics of vulnerability

We identify three key types of dynamics of vulnerability, namely underlying dynamics of vulnerability, dynamics during long-lasting vulnerability, and dynamics of vulnerability owing to consecutive or compound disasters (Figure 1). Here, we discuss each type of dynamic and provide examples of their causes. First, there can be many underlying dynamics of vulnerability at play, such as conflict, (internal) migration, or economic recession (Figure 1A). Important aspects influencing these general, non-hazard specific dynamics of vulnerability in time and space have been widely recognized in the literature and include changes in regional population and economic characteristics (Fuchs and Glade, 2016); immigration and displacement (e.g. Bronfman et al., 2021; Jayawardhan, 2017); and poverty, conflict and political (in)stability (e.g., Brooks et al., 2005). Within the African context, the IPCC et al. (2022) sixth assessment report provides a thorough overview of underlying socio-economic processes of vulnerability. Although there is a recognition and understanding of the influence of such social processes on changing vulnerability, risk assessments do not typically account for these dynamics of vulnerability (Drakes and Tate, 2022; Simpson et al., 2021). This includes the use of national vulnerability indicators that do not properly represent (changes in) local vulnerability, spatial aspects such as ripple effects of the impacts of disasters outside of the directly impacted area, and changes in vulnerability factors over time. For example, Bronfman et al. (2021) assessed social vulnerability to natural hazards throughout Chile in three time periods using SoVI and found that some districts had a stable or decreasing vulnerability, whilst others showed strongly increasing vulnerability. Not accounting for these dynamics of vulnerability could result in an over- or underestimation of risk, would disregard the increased influence of social processes in disasters, and would limit the options to prevent disasters.

Secondly, during long-lasting disasters such as droughts, crop pest infestations, and pandemics, vulnerability is likely to change over time, making pre-event values of vulnerability or averages of vulnerability over longer time periods less accurate and are likely to cause an underestimation of risk during the later phases of the event (Figure 1B). Long disasters gradually erode people’s financial resources, job opportunities, mental and physical health, social networks, and so forth. Similarly, pandemics such as COVID-19 increase unemployment (Su et al., 2021), which increases vulnerability to other hazards whilst the pandemic is still unfolding (Mishra et al., 2021). Rural populations, for example, show clear changes in vulnerability during multi-year drought, for example, increases in mental health issues (OBrien et al., 2014), financial struggles, reduced access to education, and family and community conflicts (Keshavarz et al., 2013). Vulnerability during long-lasting disasters can also change owing to changes in risk management, response, and adaptative capacity (e.g., Brooks et al., 2005; Watts et al., 2012; Kreibich et al., 2017), and changes in risk awareness and preparedness (e.g., Kreibich et al., 2017), as indicated by the spiral in Figure 1B. Also, recovery of vulnerability to pre-disaster levels after the event itself ended is important. Societies that recover their vulnerability quickly will be less vulnerable to the next event than societies that recover slowly (Di Baldassarre et al., 2018a; 2018b). Kreibich et al. (2017) found that across eight case studies in Europe, Asia and Africa reductions in vulnerability led to lower damage after a second consecutive flood event. It is important to note that risk management can have both increasing and decreasing effects on vulnerability. For example, recent studies have shown that the risk management of an individual hazard can lead to increased risk of another hazard (de Ruiter et al., 2021a, 2021b; Ward et al., 2020) or responses to climate change can have adverse effects on climate risk (IPCC, 2019; Schipper, 2020; Simpson et al., 2021). Schipper (2020) recognizes three types of climate change maladaptation and their effect on vulnerability: rebounding vulnerability (an adaptation measure aimed to decrease a group’s vulnerability makes them more vulnerable), shifting vulnerability (redistribution of vulnerability), and negative externalities (adverse effects on anyone who was not the target of a measure). In summarizing, while aspects influencing underlying dynamics of vulnerability are well understood, the assessment of these underlying dynamics of vulnerability is lacking.

Thirdly, vulnerability can change over time owing to the consecutive compounding occurrence of disasters (IPCC et al., 2022; Simpson et al., 2021) (Figure 1C). The impacts of consecutive or compounding disasters are often exacerbated by, firstly, the consecutive nature of the hazards themselves (Hillier and Dixon, 2020; Mora et al., 2018; Ridder et al., 2020; Zscheischler et al., 2018), such as wildfires following drought conditions and heatwaves (AghaKouchak et al., 2020). Secondly, spatiotemporal dynamics in exposure and vulnerability are caused by the chain of events (de Ruiter et al., 2020; Reichstein et al., 2021). For example, ash deposits on rooftops from a volcanic eruption can increase a building’s vulnerability to a subsequent earthquake (Spence et al., 1996) or a disaster can weaken the resilience of socioeconomic networks which increases vulnerability to future disasters (Kruczkiewicz et al., 2021). Hence, the impacts of an earlier event are likely to change vulnerability at the time of the next event. Nonetheless, such changes in vulnerability to a hazard owing to existing impacts from an earlier event are commonly not accounted for (Korswagen et al., 2019).

As all of these different types of dynamics can span across large spatial scales and longer time windows, it is challenging for the general public as well as policy makers to understand the complexities of these long-lasting dynamics of vulnerability (Reichstein et al., 2021) and for researchers to integrate these dynamics of vulnerability into dynamic risk assessments. Therefore, in the next section, we discuss possible methods that are commonly used in the compound and multi-hazard, and socio-hydrology fields to assess hazard (driver) dynamics, to integrate the dynamics of vulnerability into dynamic risk assessments.

## Review of methods

In more recent years, a shift can be observed from the traditional focus on single-hazard research to multi-hazard research (e.g., Gallina et al., 2016; Gill and Malamud, 2014; Kappes et al., 2012). For the assessment of dynamic vulnerability with a system perspective there is great potential in this field of compound hazards (Leonard et al., 2014; Zscheischler et al., 2018), but also in the field of socio-ecological systems (SES; Schlueter et al., 2012), sub-fields such as socio-hydrology (Sivapalan et al., 2012), and hydrosocial systems (Linton and Budds, 2014; Wesselink et al., 2017), which build on approaches developed by social science and modeling communities.

Socio-ecological systems modeling have developed from earth system science modeling (Schlueter et al., 2012; Wesselink et al., 2017) in the late 1990s and early 2000s. SES models were designed to include feedback between human society and the environment in order to capture the “co-evolution of human and natural systems” (Biermann, 2007, p. 4). In the 2010s, socio-hydrology developed as the science of human-water system dynamics (Sivapalan et al., 2012; Xu et al., 2018) with a focus on modeling these dynamics and with the water system as a starting point. This development of socio-hydrology complements a longer history of hydrosocial research (Linton and Budds, 2014), which is grounded in social science and uses more constructivist paradigms, holistic ontology, and qualitative methodology (Wesselink et al., 2017). Recent studies have shown that the co-evolution of human-water systems can lead to increased vulnerabilities, for example via changes in flood risk perception (Fuchs et al., 2017), via reservoirs that increase water dependency (Di Baldassarre et al., 2018a; 2018b), or via developments in water supply infrastructure and changes in management (Srinivasan, 2015).

A growing body of literature has discussed guidelines and methods to quantify compound hazards (e.g., Bevacqua et al., 2021) and multi-hazard relationships (e.g., Tilloy et al., 2019). In their review, Tilloy et al. (2019) define changing conditions as “one hazard changing the disposition of a second hazard by changing environmental conditions,” thereby excluding changing socio-economic conditions. Matrices have been used to identify interrelations between natural hazards (e.g., Gill and Malamud, 2016). The extensive review by Tilloy et al. (2019) demonstrates how large the growing body of literature is on methods to assess the dynamics of compound and multi-hazards. We argue that many of those methods commonly used in the SES, hydrosocial research, and compound and multi-hazard field have the potential to also be used to capture dynamics of vulnerability.

We categorized methods based on their aim. First, there are qualitative methods such as the narratives approach that can be used to get a better understanding of underlying processes. Secondly, findings from those can in turn be used to develop agent-based models (ABMs) and system dynamics models that are used to assess feedback within the system: by changing one thing, what happens to the system? Finally, the finding from those models can be used to explore the future for which scenarios and storylines are common methods used: when taking a future perspective, what are plausible scenarios? This is not a comprehensive list of methods that could be used to assess the dynamics of vulnerability. In this overview, for example, we have excluded quantitative data-based approaches, which are fully based on empirical data derived from observations. For the analysis of dynamic vulnerability, this data is often not available on the appropriate temporal and spatial scales and therefore this method has been excluded for now.

### Qualitative methods

Qualitative data help to understand the complexities of dynamic vulnerability from a systems perspective and can be gathered by a range of methods. Methods from the social sciences include interviews with local water users or managers, participant diaries, oral recollections or narrative interviews, community histories, participant observation, photographs, and other visual or text-based materials (Rangecroft et al., 2021). The advantage of these methods is that political, cultural, and economic context is taken into account.

Narrative interviews, also known as qualitative storylines, are especially powerful to assess dynamic vulnerability as changes in time and space play a prominent role in this method. They are commonly used in social sciences (Jones et al., 2014) but in recent years have also been used in climate change communication (Shepherd et al., 2018). Narratives can be thought of as stories based on past personal experiences (Schacter et al., 2007; Tulving, 1972). When conducting a structured or semi-structured interview, the interviewer is likely to receive answers bounded by the question asked. Narrative interviews instead revolve around the question “What happened when, where, and why?” and give more power to the participant to shape the story, include different perspectives and emotions, and changes in time (McBeth et al., 2014; Squire, 2013). Recent studies have used narrative methods to study the lived experiences of droughts and floods, giving some insights also in the temporal and spatial dynamics of vulnerability (e.g. Greene, 2021; Quandt, 2021; Bryan et al., 2020; Malik & Najmul Islam Hashmi, 2020). For example, stories of lived experiences of drought show that vulnerabilities change over long timescales and across groups in society (Greene, 2021), while coping strategies can reduce vulnerabilities (Quandt, 2021), exemplifying the effects in Figure 1B. Malik and Najmul Islam Hashmi (2020) show how historical social and political-economic processes increase vulnerability to floods, exemplifying the effects in Figure 1A. We see more potential in these methods, especially to complement more quantitative methods of the dynamics of vulnerability. Qualitative data such as collected via the narratives approach can also be used as the basis of models.

### Agent-based and system dynamics models

ABMs and system dynamic models are commonly used models to understand processes and the effects of feedback within a system (Blair and Buytaert, 2016). System dynamics (SD) models use a top-down approach to simulate overall system behavior based on the understanding of the processes connecting system components. In these models, social aspects such as vulnerability, awareness, and memory that are hard to quantify or measure play a key role. These are important to understand non-linearities and surprises in the coupled system. For example, subsequent droughts can make a society vulnerable until they reach a tipping point (e.g. Kuil et al., 2016, who modeled the Maya collapse). The development of SD models starts with a conceptualization of the human-water system in causal loop diagrams, which are then converted into a set of nonlinear differential equations (Blair and Buytaert, 2016; Joakim et al., 2016). In some recent studies, SD models have been used to explicitly include social vulnerability in hazard and risk research (Joakim et al., 2016; Zarghami and Dumrak, 2021). These studies show that in SD social vulnerability is an integral part of the system and it changes when other components of the system change, leading to both reducing and exacerbating vulnerability, as well as the generation of new vulnerabilities. The literature review by Joakim et al. (2016) indicated that SD modeling did not have a strong role in the disaster management literature, but that there is a large potential.

Recent years have seen a flight in the development of ABMs to model human behavior before, during, and after a disaster (Aerts et al., 2018; Schrieks et al., 2021). ABMs consist of agents, the environment, and a temporal component, and they are able to represent collective social dynamics (Terzi et al., 2019); for example, the impacts of a disaster or a policy on the system (Klabunde and Willekens, 2016). ABMs are used to model individual agents and their decision-making process, interactions with other agents, and the effects of those interactions on their own behavior, to assess the dynamics of a social network over time and over space (Klabunde and Willekens, 2016). This in turn can be used to support spatial predictions. ABMs require empirical data for estimation and for validation. Therefore, the use of an ABM allows for the modeling of migration patterns that result from consecutive disasters under current and future conditions. Agent-based models were excluded from Tilloy et al. (2019) as the authors deemed this method weaker in terms of its ability to address uncertainties. Although recognizing the inability of ABMs to assess uncertainty, others have discussed the successful use of ABMs and/or system dynamic models to assess hazard or risk dynamics (Johnson, 2005; Terzi et al., 2019). Terti et al. (2015) argue that ABMs could be particularly useful in modeling the influence of cognitive processes on crisis behavior as a source of spatial and temporal dynamics in the vulnerability of individuals, for example during a flash flood.

### Scenarios and storylines

The storylines approach (Shepherd et al., 2018) has been receiving growing attention from the compound hazard community (e.g., Matthews and Marston, 2019). This approach has increasingly been used by the climate change community as it provides an alternative approach to representing uncertainty. Storylines allow for a deterministic, event-centric rather than a probabilistic way of framing risk (Shepherd et al., 2018). As storylines are often developed at a national, continental, or global scale, a limitation in the current use of storylines is the inability to capture detailed vulnerability. Studies have, therefore, recommended developing ways of focusing on sub-national scales (e.g., (Vervoort et al., 2014). Although it is currently not often conducted, or only with a focus on physical/infrastructure vulnerability (e.g. De Bruijn et al., 2016), vulnerability can be included explicitly in a storyline approach (Sillmann et al., 2021). Storylines can be developed using different model approaches including SD models. Besides quantitative storylines, qualitative storylines based on narratives can be developed as thought experiments for future scenarios.

Both qualitative and quantitative scenarios also allow for the modeling of possible futures, ideally including the interplay between human and environmental systems using SES models (Elsawah et al., 2020). The scenario approach is based on the idea that while the future is uncertain, it is not entirely unknowable. Modeling these scenarios is not meant to predict the future, but rather to explore and compare a range of different, plausible futures and pathways to get to these futures (Lempert, 2013). When conducted with SES approaches or socio-hydrological modeling, this can be used to assess different conditions that contribute to exacerbating vulnerability (Elsawah et al., 2020). Scenarios have been used in the assessment of vulnerability (especially for context-specific SES focusing on a particular geographical area of an individual sector and particularly to support decision making (e.g., Absar and Preston, 2015; Carlsen et al., 2016; Lempert, 2013) and can account for the dynamics of risk (Riddell et al., 2019).

## Conclusions

As the release of the SFDRR, there has been a push toward a better understanding of the dynamics of risk. In recent years, this has led to an increase in research by the compound and multi-hazard community looking into the dynamics of hazards. A less well-understood component of risk is that of the dynamics of vulnerability.

Around the turn of the century, researchers acknowledged the complexities of vulnerability, especially when widening the lens from physical to social vulnerability. We discuss several key developments in the field of vulnerability research in subsequent years, including (1) changes in common indicators used to measure vulnerability, (2) a shift from a focus on physical vulnerability to including social vulnerability, and (3) most recently a change from static toward dynamic vulnerability. To complicate things furthermore, more recently, it became clear that the line between social processes as a hazard versus as a source of vulnerability is blurred.

We identify three dynamics of vulnerability that are often excluded from dynamic risk assessments: (1) the underlying dynamics of exposure and vulnerability, (2) changes in vulnerability during long-lasting disasters, and (3) changes in vulnerability during compounding disasters. These different types of dynamics can span across large spatial scales and long-time windows. This contributes to the challenges for the general public as well as policy makers to understand the complexities of these long-lasting dynamics of vulnerability and for researchers to integrate these dynamics of vulnerability into dynamic risk assessments. Therefore, we discuss possible methods that are commonly used in the fields of compound hazards, but also in the field of socio-ecological systems, sub-fields such as socio-hydrology, and hydrosocial systems.

In these fields, the growing attention for research into the dynamics of hazard (drivers) has also caused a surge in the application of (for this field) novel methods, including agent-based models, scenarios and storylines, and qualitative methods. We argue that many of these methods offer great potential to capture the dynamics of vulnerability. We identify three method categories based on their aim: (1) qualitative methods such as the narratives approach that can be used to get a better understanding of underlying processes; (2) agent-based models (ABMs) and System Dynamics, which can use the findings from qualitative assessments; and (3) scenarios and storylines, which can use the findings from ABMs and System Dynamic models to explore plausible future scenarios. Although our article focused on vulnerability from a disaster risk perspective, our suggestions can expand to the broader field of vulnerability research.
